# Study on the Protective Effect and Mechanism of the Rhizoma Drynariae-Epimedium Formula on Osteoarthritis in Rats

**DOI:** 10.1155/2022/2869707

**Published:** 2022-05-29

**Authors:** Zonghui Dai

**Affiliations:** Yangtze University Health Science Center, Jingzhou 434100, China

## Abstract

**Purpose:**

The aim of the study was to study the protective effect of the Rhizoma Drynariae-Epimedium formula on osteoarthritis in rats and to explore its mechanism.

**Methods:**

Fifty SD rats were randomly divided into 5 groups, namely, the control group, model group, Rhizoma Drynariae group, Epimedium group, and Rhizoma Drynariae-Epimedium group, with 10 rats in each group. Knee arthritis models were established by injecting papain solution (10% papain + 0.03 mol/L L-cysteine mixture) into the knee joint cavity of SD rats on the 0th, 3^rd^, and 6th days of the experiment, respectively. The model group, Rhizoma Drynariae group, Epimedium group, and Rhizoma Drynariae-Epimedium group were given modeling treatment, while the control group was not given modeling treatment. The Rhizoma Drynariae group, Epimedium group, and the Rhizoma Drynariae-Epimedium group were, respectively, given corresponding solvent gavage treatment. Both the model group and the control group were given an equal volume of normal saline. Once a day, a total of 4 w were administered. The general conditions of the rats were observed and recorded, and the knee joint width and the knee joint swelling degree of the affected side were measured and compared. HE staining and Safranin O-fast green staining were used to compare the structural changes of cartilage. The concentrations of inflammatory factors IL-1*β*, IL-6, and TNF-*α* in the joint cavity lavage fluid were determined by using ELISA. The expression of key proteins of the MAPK signaling pathway (p38, p-p38, ERK, p-ERK, JNK, and p-JNK) in joint synovial tissue was determined by western blotting.

**Results:**

After modeling, except for the normal activities of the SD rats in the control group, the rest of the groups showed lack of energy and a slight limp in the knee joints. The SD rats in the model group, Rhizoma Drynariae group, Epimedium group, and Rhizoma Drynariae-Epimedium group had local swelling of the knee joint, and the knee joint width was greater than those in the control group (*p* < 0.05). Compared with the model group, the knee joint swelling of SD rats in the Rhizoma Drynariae group, the Epimedium group, and the Rhizoma Drynariae-Epimedium group was significantly reduced. The knee joint swelling degree of SD rats in the Rhizoma Drynariae-Epimedium group was significantly lower than that in the Rhizoma Drynariae and Epimedium groups. HE staining and Safranin O-fast green staining showed that the cartilage structure of SD rats was severely damaged and eroded, and the subchondral bone mass was reduced. Compared with the model group, the damage of cartilage tissue in the Rhizoma Drynariae group, Epimedium group, and Rhizoma Drynariae-Epimedium group was less severe. In the Rhizoma Drynariae-Epimedium group, cartilage tissue structure damage and erosion were lighter than those of the Rhizoma Drynariae group and the Epimedium group. The concentrations of inflammatory factors IL-1*β*, IL-6, and TNF-*α* in the articular cavity lavage fluid of SD rats in the model group, Rhizoma Drynariae group, Epimedium group, and Rhizoma Drynariae-Epimedium group were higher than those in the control group. Compared with the model group, the concentrations of IL-1*β*, IL-6, and TNF-*α* in the joint cavity lavage fluid of the Rhizoma Drynariae group, Epimedium group, and Rhizoma Drynariae-Epimedium group were significantly decreased. In the Rhizoma Drynariae-Epimedium group, IL-1*β*, IL-6, and TNF-*α* concentrations were lower than those of the Rhizoma Drynariae and Epimedium groups. Compared with the control group, the expression levels of p-p38, p-ERK, and p-JNK proteins in the model group, Rhizoma Drynariae group, Epimedium group, and Rhizoma Drynariae-Epimedium group were significantly increased. The expression levels of p-ERK, p-p38 and p-JNK in the Drynariae group, Epimedium group, and Drynariae-Epimedium group were significantly lower than those in the model group. The expression levels of p-ERK, p-p38, and p-JNK in the Rhizoma Drynariae-Epimedium group were significantly lower than those in the Rhizoma Drynariae and Epimedium groups.

**Conclusion:**

The Rhizoma Drynariae-Epimedium formula can play a protective role in the process of osteoarthritis by inhibiting the phosphorylation levels of p38, ERK, and JNK-related proteins in the cartilage tissue MAPK signaling pathway, reducing the inflammatory response.

## 1. Introduction

Osteoarthritis (OA) is a common chronic, degenerative joint disease. It occurs in middle-aged and elderly people, and the incidence is proportional to age [1]. It is clinically characterized by destruction of articular cartilage, pain, and stiffness, which ultimately lead to immobility and the reduced quality of life for patients, thereby increasing the social and medical burden [2]. Artificial joint replacement is mainly suitable for advanced patients with severe symptoms, but there is still a lack of substantial progress in the treatment of early and midstage patients [3]. In-depth study of the pathogenesis of osteoarthritis has important clinical significance for improving the level of diagnosis and treatment of osteoarthritis. At present, the use of proprietary Chinese medicines to treat osteoarthritis has become a hot topic in the academic circles [4]. Rhizoma Drynariae, as a prescription in the treatment of osteoarthritis, has the effect of nourishing the liver and kidney, strengthening the muscles and bones [5]. Epimedium has the functions of invigorating the kidney and yang, strengthening the muscles and bones, and is a commonly used medicine in traditional Chinese medicine to prevent and treat osteoarthritis [6]. Although both Rhizoma Drynariae and Epimedium have certain benefits in the treatment of osteoporosis [7], the application value and mechanism of Rhizoma Drynariae-Epimedium in osteoarthritis are rarely reported. The purpose of this study was to establish the SD rat model of osteoarthritis, to study the protective effect of the Rhizoma Drynariae-Epimedium formula on rat osteoarthritis, and to explore its possible mechanism of action.

## 2. Materials and Methods

### 2.1. Experimental Materials

Papain (Article No.: P3250, Sigma), L-cysteine (Article No.: C0012-25, Beijing Solebo Technology Company), EDTA decalcification liquid (Article No.: B1056-100, Beijing Pleilai Co., LTD.), mouse interleukin-1*β* (IL-1*β*) ELISA Kit E-el-m0037c, Wuhan Ilerite Biological Co., LTD.), interleukin-6 (IL-6) (ELISA Kit Zc-37988, Shanghai Zhuocai Biological Company), tumor necrosis factor-*α* (TNF-*α*) (ELISA Kit Jl10484-48t, Shanghai Jianglai Biological Company), extracellular signal-regulated kinase (ERK) primary antibody (Article No.: AB242993, Abcam Company), p38 primary antibody (Article No.: Ab178867, Abcam), C-Jun N-terminal kinases (JNKs) primary antibody (Article No.: AB76125, Abcam), p-JNK primary antibody (Article No.: Ab219584, Abcam), p–p38 primary antibody (Article No.: AB240335, Abcam), p-ERK primary antibody (Article No.: AB214036, Abcam), GAPDH primary antibody (Article No.: Ab8245, Abcam Company), and sheep antirat second antibody were purchased from Wuhan Baishi Biological Company; western blotting kit (Wuhan Baishi Biological Company) and Bone Sububu and Herba medium were purchased from Anhui Zhengtang Traditional Chinese Medicine Drinks Co., LTD. Fifty male SD rats (SPF grade), weighing 250 ± 20 g, were purchased from experimental Animal Center of China Three Gorges University (License No. SCXK (E) 2017-0012).

### 2.2. Animals

Animal male BALB/c rats (10 weeks; 300–350 g) were provided by the Center for Animal Experiment of China Three Gorges University (Yichang, China). Mice were housed for at least 1 week in specific pathogen-free conditions, with a 12 h light/dark cycle prior to the experiment and unrestricted access to food and water. All experimental procedures were conducted in accordance with the Guide for the Care and Use of Laboratory Animals (National Institutes of Health, Bethesda, MD, USA). The study protocol was also approved by the Animal Ethics Committee of Yangtze University (Jingzhou, China).

### 2.3. Establishment of Animal Models

The SD rats were randomly divided into 5 groups, namely, the control group, model group, Rhizoma Drynariae group, Epimedium group, and Rhizoma Drynariae-Epimedium group, with 10 rats in each group. The modeling steps were as follows: intravenous injection of papain solution (10% papain + 0.03 mol/L L-cysteine mixture) into the knee joint cavity of SD rats to establish a knee arthritis model [8]. The rat model of knee arthritis was induced by injecting 0.2 mL of the modeling solution into the knee joint cavity of SD rats on the 0th, 3rd, and 6th days of the experiment, respectively. The model group, Rhizoma Drynariae group, Epimedium group, and Rhizoma Drynariae-Epimedium group were given modeling treatment, while the control group was not given modeling treatment. After the last intra-articular injection of the knee joint, the Rhizoma Drynariae group, the Epimedium group, and the Rhizoma Drynariae-Epimedium group were, respectively, given the corresponding water decoction solution by gavage, and the gavage dose was 1.5 g/kg [9]. Both the model group and the control group were given an equal volume of normal saline. Once a day, a total of 4 w were administered. The general conditions of the rats were observed and recorded, and the rats were euthanized 4 weeks after modeling; the joint cavity lavage fluid, cartilage tissue, and synovial tissue of 5 groups were collected and stored in a −80°C refrigerator for testing.

### 2.4. Measurement of Joint Width and Joint Swelling in Rats

The right lower limb of the rat was stretched, the knee joint was exposed, and a vernier caliper was used to measure along the widest part of the knee joint, and the values were recorded before and 4 weeks after modeling. Calculation of the knee joint swelling degree: knee joint swelling degree = (joint width after 4 weeks − joint width before modeling)/joint width before modeling × 100%.

### 2.5. Histopathological Testing

The right knee joint was taken, and the extracted cartilage tissue was placed in an embedding box and fixed in formaldehyde for 24 h. After rinsing with clean water, decalcify 1 m with EDTA decalcification solution, and it is qualified if the needle can be inserted into the bone tissue specimen. Then, dehydration with gradient alcohol, clearing with xylene, dipping in wax, embedding, and sectioning were performed in sequence. Standard hematoxylin-eosin staining and Safranin O-fast green staining were performed, and histopathological changes were observed by light microscopy.

### 2.6. Detection of Inflammatory Factor Expression in the Joint Cavity Lavage Fluid Using ELISA

According to the instructions of the ELISA kit, the levels of interleukin-1*β* (IL-1*β*), interleukin-6 (IL-6), and tumor necrosis factor-*α* (TNF-*α*) in the lavage fluids of the five groups were measured. The microplate reader is set to a wavelength of 450 nm, the OD value of each well is detected, and a standard curve is established to obtain the conversion formula between the concentration and the OD value, and the results are calculated based on this.

### 2.7. Determination of MAPK Signaling Pathway Protein Expression by Western Blot

The joint synovial tissue of the control group, model group, Rhizoma Drynariae group, Epimedium group, and Rhizoma Drynariae-Epimedium group was placed on ice, and 150 *μ*L of RIPA lysis solution containing protease inhibitor was added, and the synovial tissue was fully ground to a homogenate. The protein concentration was detected by the BCA method; then, a 5x buffer was added and denatured in a 100°C metal thermostatic incubator for 10 min. A 10% SDS-PAGE gel was prepared, and electrophoresis after protein loading was run. The constant pressure is first 80 V for 30 min and then transferred to 100 V for 2 h. When the bromophenol blue runs to the end, it is completed. Subsequently, the gel was cut and transferred into the PVDF membrane. After blocking with 5% nonfat milk for 1 hour at room temperature, incubate with the following specific antibodies for 16 hours at 4°C : ERK primary antibody (1 : 300), p38 primary antibody (1 : 300), JNK primary antibody (1 : 200), p-JNK primary antibody (1 : 200), p–p38 primary antibody (1 : 300), p-ERK primary antibody (1 : 300), and GAPDH primary antibody (1 : 500). After incubation, the PVDF membrane was rinsed with the TBST buffer for 10 min and rinsed 3 times. Then, a goat antimouse secondary antibody (1 : 800) was added and incubated at room temperature for 1 h, followed by rinsing with the TBST buffer for 10 min and rinsing 3 times. ECL solution was added to the PVDF membrane and exposed in the dark room, and the gray value of the target band was calculated with ImageJ software; target protein expression = gray value of target band/gray value of GAPDH band.

### 2.8. Statistical Analysis

Statistical analysis was performed using Prism GraphPad 8.0 software, and measurement data were expressed as the mean ± standard deviation (mean ± SD). One-way ANOVA was used for the comparison between multiple groups. On the basis of statistical significance, the LSD-T test was used for the comparison between the two groups. *p* < 0.05 was considered to be statistically significant.

## 3. Result

### 3.1. Rhizoma Drynariae-Epimedium Decoction Improves Osteoarthritis Symptoms

**Figure 1:**
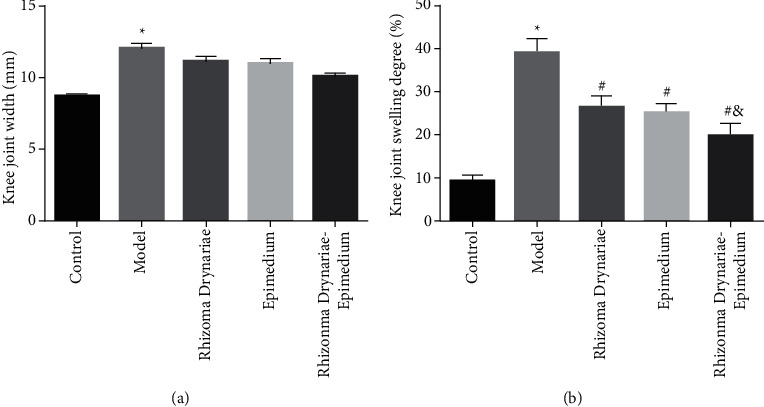
Comparison of the knee joint width and knee joint swelling degree of SD rats in 5 groups. (a) Knee joint width. (b) Knee joint swelling degree. Data are presented as mean ± SD (*n* = 10 for each group). ^*∗*^*p* < 0.05 versus the control group; ^#^*p* < 0.05 versus the model group; ^&^*p* < 0.05 versus the Rhizoma Drynariae group and the Epimedium group. Three independent experiments were performed.

General conditions of rats: before the experiment, SD rats in the 5 groups had a normal range of motion of knee joints, no local swelling, smooth fur, good sensitivity, normal mental state, and ability to eat and drink. SD rats did not die during the modeling process. After modeling, except for the normal activities of SD rats in the control group, the other groups showed different degrees of lack of energy and limping of the knee joint. SD rats in the model group, Rhizoma Drynariae group, Epimedium group, and Rhizoma Drynariae-Epimedium group had local swelling of the knee joint, and the knee joint width was greater than that in the control group. Compared with the model group, the knee joint swelling of SD rats in the Rhizoma Drynariae group, Epimedium group, and Rhizoma Drynariae-Epimedium group was significantly decreased. The knee joint swelling degree of SD rats in the Rhizoma Drynariae-Epimedium group was significantly lower than that in the Rhizoma Drynariae and Epimedium groups (Figure 1).

### 3.2. Rhizoma Drynariae-Epimedium Decoction Inhibits Cartilage Tissue Damage

**Figure 2:**
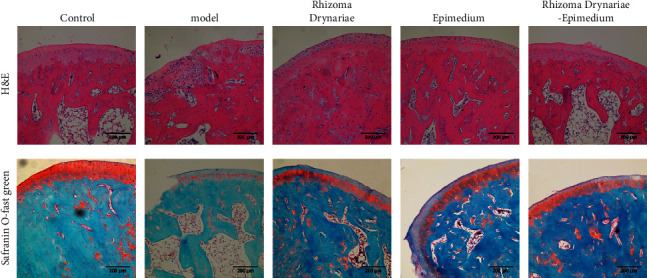
HE staining and Safranin O-fast green staining of rat knee joints of SD rats in five groups to evaluate histopathological changes.

HE staining and Safranin O-fast green staining showed that after the injection of papain solution into the joint cavity, the cartilage structure of SD rats was severely damaged and eroded, and the subchondral bone mass was reduced. Compared with the model group, the damage of cartilage tissue in the Rhizoma Drynariae group, Epimedium group, and Rhizoma Drynariae-Epimedium group was less severe. The damage and erosion of the cartilage tissue structure in the Rhizoma Drynariae-Epimedium group were lighter than those in the Rhizoma Drynariae group and the Epimedium group (Figure 2).

### 3.3. Rhizoma Drynariae-Epimedium Decoction Reduces Joint Inflammation

**Figure 3:**
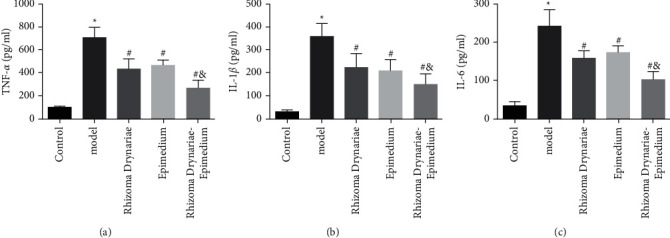
Comparison of the concentrations of inflammatory factors IL-1*β*, IL-6, and TNF-*α* in the knee joint lavage fluid of SD rats in five groups. (a) TNF-*α*. (b) IL-1*β*. (c) IL-6. Data are presented as mean ± SD (*n* = 10 for each group). ^*∗*^*p* < 0.05 versus the control group; ^#^*p* < 0.05 versus the model group; ^&^*p* < 0.05 versus the Rhizoma Drynariae group and the Epimedium group. Three independent experiments were performed.

ELISA results showed that the concentrations of inflammatory factors IL-1*β*, IL-6, and TNF-*α* in the joint cavity lavage fluid of SD rats in the model group, Rhizoma Drynariae group, Epimedium group, and Rhizoma Drynariae-Epimedium group were higher than those in the control group. Compared with the model group, the concentrations of IL-1*β*, IL-6, and TNF-*α* in the joint cavity lavage fluid of the Rhizoma Drynariae group, Epimedium group, and Rhizoma Drynariae-Epimedium group were significantly decreased. The Rhizoma Drynariae-Epimedium group had lower concentrations of IL-1*β*, IL-6, and TNF-*α* than the Rhizoma Drynariae and Epimedium groups (Figure 3).

### 3.4. Effects of Rhizoma Drynariae-Epimedium Decoction on the MAPK Signaling Pathway

**Figure 4:**
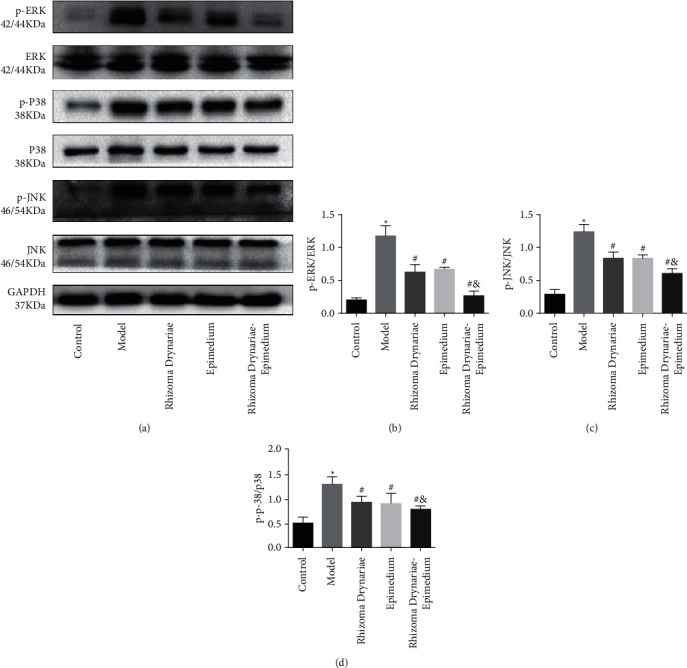
Expression of the MAPK signaling pathway protein in knee joint synovial tissue of five groups of SD rats detected by western blotting. (a) Western blot. (b) p-ERK/ERK. (c) p-P38/P38. (d) p-JNK/JNK. Data are presented as mean ± SD (*n* = 10 for each group). ^*∗*^*p* < 0.05 versus the control group; ^#^*p* < 0.05 versus the model group; ^&^*p* < 0.05 versus the Rhizoma Drynariae group and the Epimedium group. Three independent experiments were performed.

Western blotting experiments on joint synovial tissue of SD rats in 5 groups showed that compared with the control group, the expressions of p-p38, p-ERK, and p-JNK in the model group, Rhizoma Drynariae group, Epimedium group, and Rhizoma Drynariae-Epimedium group were significantly increased. The expression levels of p-ERK, p-p38, and p-JNK in the Rhizoma Drynariae group, Epimedium group, and Rhizoma Drynariae-Epimedium group were significantly lower than those in the model group. The expression levels of p-ERK, p-p38, and p-JNK in the Rhizoma Drynariae-Epimedium group were significantly lower than those in the Rhizoma Drynariae and Epimedium groups (Figure 4).

## 4. Discussion

The theory of traditional Chinese medicine believes that “the kidney controls the bone and generates the marrow, which is the foundation of the innate,” and the strength of the bones is closely related to the filling of the kidney essence. Rhizoma Drynariae and Epimedium are commonly used prescriptions in the Department of Orthopedics and Traumatology of Traditional Chinese Medicine. Rhizoma Drynariae is the dry rhizome of Quercus quercus, a plant of the family Hydrangea, and its chemical constituents mainly include flavonoids, triterpenes, phenylpropanoids, and lignans [10]. The main active ingredient of Rhizoma Drynariae is total flavonoids. Total flavonoids can not only promote the proliferation of osteoblasts, reduce the activity of osteoclasts, increase bone density, inhibit bone resorption, and promote the healing of bone defects [11], but also have analgesic, lipid-regulating, and anti-inflammatory effects [12]. Epimedium is a traditional herbal medicine that has been used for centuries, and its main active ingredient is icariin, a prenylated flavonol glycoside [13]. Icariin can promote the formation of osteoblasts and activate the activity of osteoblasts, promote the transformation of bone marrow-derived mesenchymal stem cells into osteoblasts, promote the maturation of preosteoblasts, and inhibit the differentiation of primary osteoblasts into adipocytes [14]. In addition, icariin can inhibit bone resorption by inhibiting osteoclastogenesis, thereby inhibiting the biological activity of osteoclasts [15] and can also reduce bone loss by inhibiting inflammatory signaling pathways [16]. The two are often used as prescriptions and are often used clinically to treat rheumatism, fractures, bone defects, bone and joint diseases, osteoporosis, and gonadal dysfunction and have achieved good clinical results [17]. Wang et al. reported that the total flavonoids in Rhizoma Drynariae can induce changes in bone morphology, expression of the vascular endothelial growth factor and CD31, and promote bone healing and reconstruction [18]. Chen et al. reported that total flavonoids in Rhizoma Drynariae can promote the angiogenesis-osteogenesis coupling process in bone defect reconstruction by activating the notch pathway and ultimately promote the repair of defective bone [19]. Ding et al. reported that the total flavonoids in Rhizoma Drynariae can promote the proliferation of rat bone marrow mesenchymal stem cells and the differentiation of osteoblasts by upregulating the expressions of *β*-catenin and RunX2 and inhibiting and downregulating the expression of PPARG [20]. Yeung et al. reported that total flavonoids in Rhizoma Drynariae could inhibit matrix metalloproteinase 3 and TNF-*α* in SD rats, inhibit NF-kB signaling pathway, and then delay intervertebral disc degeneration [21]. It has also been reported in the literature that icariin can promote bone formation in SD rats with tibial fractures, and the expression of protein 2, vascular growth factor, Runt-related transcription factor 2, and alkaline phosphatase, thereby increasing the bone mineral density at the fracture site and promoting tibial healing [22].

Based on this, the author speculates that Rhizoma Drynariae-Epimedium may have a certain therapeutic effect on osteoarthritis. In this study, a papain-induced SD rat knee osteoarthritis model was established and SD rats were treated with decoction of Rhizoma Drynariae, Epimedium, and the Rhizoma Drynariae-Epimedium formula by gavage, respectively. All rats were observed for knee arthritis symptoms, pathological damage, inflammatory factor expression, and MAPK signaling pathway-related protein expression. After modeling, the knee joint swelling width and knee joint swelling degree were observed. It was found that SD rats in the model group, Rhizoma Drynariae group, Epimedium group, and Rhizoma Drynariae-Epimedium group had local swelling of the knee joint, and the degree of knee joint swelling was greater than that of the control group, indicating that the modeling of knee osteoarthritis was successful. HE staining and Safranin O-fast green staining showed that the damage and erosion of the cartilage structure of SD rats in the model group were severe, and the subchondral bone mass was reduced, while the damage and erosion of the cartilage tissue structure in the Rhizoma Drynariae-Epimedium group were lighter than those in the Rhizoma Drynariae and Epimedium groups. It is suggested that the Rhizoma Drynariae-Epimedium formula have a certain therapeutic effect on knee arthritis in SD rats, and the Rhizoma Drynariae-Epimedium group may play a synergistic therapeutic effect.

IL-1*β*, IL-6, and TNF-*α* are common cellular inflammatory factors, which are often used to reflect the inflammatory state of the body [23]. In this study, the expression of inflammatory factors in the SD rat knee lavage fluid was further determined. It was found that the concentrations of IL-1*β*, IL-6, and TNF-*α* in the joint cavity lavage fluid of the Rhizoma Drynariae group, Epimedium group, and Rhizoma Drynariae-Epimedium group were significantly lower than those of the control group; moreover, the Rhizoma Drynariae-Epimedium group was lower than the Rhizoma Drynariae group and the Epimedium group. These results suggest that the Rhizoma Drynariae-Epimedium formula can reduce the degree of inflammation of knee arthritis.

Inflammatory stimulation leads to the progressive loss of cartilage components, the destruction of cartilage structure and function, and the degradation of extracellular matrix, which are the main pathological changes of osteoarthritis [24]. Among them, the MAPK signaling pathway plays an important role in regulating the inflammatory response of bone and joint [25]. MAPKs are a class of serine/threonine protein kinases that are ubiquitous in eukaryotic cells. When the body is subjected to external stimuli such as trauma, inflammation, and bacterial infection, it will immediately initiate signal transduction, and it will be amplified step by step, affecting the transcription and expression of nuclear-related genes and finally causing a series of stress responses in cells [26]. The MAPK pathway has 4 main branch routes: ERK, JNK, p38, and ERK5 [27]. Among them, the JNK, p38, and ERK signaling pathways are all related to the inflammatory response. Under the stimulation of proinflammatory factors (TNF-*α*, IL-1, and IL-6 ) and stress stimulation (H_2_O_2_, heat shock, hypertonic and protein synthesis inhibitors, etc.), it can induce phosphorylation of JNK, p38, and ERK, stimulate JNK, p38, and ERK-related signaling pathways, and induce the activation of endogenous immune cells such as monocytes, endothelial cells, and neutrophils, thereby promoting the progression of inflammation [28]. In the inflammatory state, inflammatory factors such as IL-1*β*, IL-6, and TNF-*α* cause osteoclast differentiation and dysfunction through the RANK signaling pathway, resulting in excessive bone resorption, progressive loss of cartilage components, and destruction of the cartilage structure. This is the basic pathological feature of osteoporosis, arthritis, and other diseases. This study further investigates the changes in the JNK, p38, and ERK signaling pathways. It was found that the expressions of p-JNK, p-p38, and p-ERK in the Rhizoma Drynariae group, the Epimedium group, and the Rhizoma Drynariae-Epimedium group were all downregulated, and the JNK, p38, and ERK signaling pathways were inhibited; the reduction was more significant in the Rhizoma Drynariae-Epimedium group. These results suggest that the mechanism of mild inflammatory response and small knee injury in the Rhizoma Drynariae-Epimedium group may be related to inhibition of the JNK, p38, and ERK signaling pathways. Indeed, because this study is an animal experiment, the protective effect and mechanism of the Rhizoma Drynariae-Epimedium formula on knee cartilage cells have not been further elucidated at the cytological level, which deserves further study.

In summary, this study found that the Rhizoma Drynariae-Epimedium formula inhibits the release of inflammatory factors downstream of the pathway by inhibiting the phosphorylation levels of MAPK signaling pathway-related proteins p38, ERK, and JNK in cartilage tissue and exerts a protective role in the process of osteoarthritis. This will help further elucidate the mechanism of the Rhizoma Drynariae-Epimedium formula in the treatment of osteoarthritis and provide new ideas for drug research and development based on traditional Chinese medicine.

## Data Availability

The data used to support the findings of this study are available from the author upon request.
